# New Multitarget Hybrids Bearing Tacrine and Phenylbenzothiazole Motifs as Potential Drug Candidates for Alzheimer’s Disease

**DOI:** 10.3390/molecules24030587

**Published:** 2019-02-07

**Authors:** Rajeshwari Rajeshwari, Karam Chand, Emanuel Candeias, Sandra M. Cardoso, Sílvia Chaves, M. Amélia Santos

**Affiliations:** 1 Centro de Química Estrutural, Instituto Superior Técnico, Universidade de Lisboa, Av. Rovisco Pais 1, 1049-001 Lisboa, Portugal; rajeshwarichoudhary82@gmail.com (R.R.); kc4chemistry@gmail.com (K.C.); silvia.chaves@tecnico.ulisboa.pt (S.C.); 2 CNC—Center for Neuroscience and Cell Biology, University of Coimbra, 3004-504 Coimbra, Portugal; emanuel.candeias@gmail.com (E.C.); cardoso.sandra.m@gmail.com (S.M.C.); 3 Institute of Molecular and Cell Biology, Faculty of Medicine, University of Coimbra, 3004-504 Coimbra, Portugal

**Keywords:** Alzheimer’s disease, multitarget, tacrine hybrids, benzothiazole, acetylcholinesterase inhibitors, Aβ aggregation

## Abstract

Research on neurodegenerative brain disorders, namely the age-dependent Alzheimer’s disease (AD), has been intensified in the last decade due to the absence of a cure and the recognized increasing of life expectancy for populations. To address the multifactorial nature and complexity of AD, a multi-target-directed ligand approach was herein employed, by designing a set of six selected hybrids (**14**–**19**) that combine in the same entity two pharmacophores: tacrine (TAC) and 2-phenylbenzothiazole (PhBTA). The compounds contain a methoxy substituent at the PhBTA moiety and have a variable length linker between that and the TAC moiety. The docking studies showed that all the compounds assure a dual-binding mode of acetylcholinesterase (AChE) inhibition, establishing π-stacking and H-bond interactions with aminoacid residues at both active binding sites of the enzyme (CAS and PAS). The bioassays revealed that the designed compounds display excellent AChE inhibitory activity in the sub-micromolar range (0.06–0.27 μM) and moderate inhibition values for amyloid-β (Aβ) self-aggregation (27–44.6%), compounds **14** and **15** being the lead compounds. Regarding neuroprotective effects in neuroblastoma cells, compounds **15**, **16** and **19** revealed capacity to prevent Aβ-induced toxicity, but compound **16** showed the highest neuroprotective effect. Overall these hybrid compounds, in particular **15** and **16**, with promising multitarget anti-AD ability, encourage further pursuing studies on this type of TAC-PhBTA derivatives for potential AD therapy.

## 1. Introduction

Alzheimer’s disease (AD) is a severe age-dependent neurodegenerative brain disorder associated with progressive memory loss and decrease of cognitive functions. The absence of a cure so far and the expected dramatic incidence increase in the coming years, due to the population aging, place a huge burden on society, which has accounted for the current intensive studies aimed at preventing and treating AD [1,2].

Extracellular deposition of neurotoxic beta-amyloid (Aβ) plaques in the brain has been recognized as the central histological characteristic of AD, but the origin of these insoluble neurotoxic deposits is not clear and multiple factors such as metal ion dyshomeostasis and elevated oxidative stress have been reported to trigger their formation. The development of a cure for AD has been hindered by the complexity of the disease and lack of understanding of its genesis and progression [3]. Although numerous clinical trials have been explored over the last two decades for potential pharmacological treatment [4], only four acetylcholinesterase inhibitors (AChEi) (tacrine, donepezil, rivastigmine, galantamine) and memantine (antagonist of NMDA receptors) have been FDA approved as anti-AD drugs [5]. However, these are just symptomatic drugs, as they can only lead to temporary amelioration of memory and cognitive problems, but, unfortunately, they do not interfere with the course of the disease [3].

Thus, to address the multifactorial origin and inherent complexity of AD, the search for new drugs has been based on the multi-target-directed ligand (MTDL) approach, by combining in the same molecular entity, different pharmacophores able to interact with different targets [6,7]. Under this strategy, the most common structures developed in the last ten years have been inspired by existing drugs already used in therapy (i.e., tacrine, donepezil), to compensate for the massive loss of cholinergic activity, and include other pharmacophore groups to interfere with some of the classical targets, namely inhibition of amyloid-β aggregation, inhibition of tau-hyperphosphorylation, modulation of oxidative stress, chelation of misplaced metal ions, as well as the inhibition of other enzymes (secretases, monoamineoxidases) [8,9,10,11,12].

As a part of our research, an identical approach has been pursued for the development of new multi-targeting compounds as potential anti-AD agents, most of them including metal modulating chemical tools [13,14,15]. In the current study, a set of new novel tacrine-(2-phenylbenzothiazole) (TAC-PhBTA) hybrids were designed to meet the structural requirements for the inhibition of AChE and Aβ aggregation, along with the expected neuroprotection role. Although tacrine has been discontinued from the market in several countries, due to its side effects at therapeutic doses [9], it has been by far the most used moiety in the repositioning of AChEi drugs, hoping that the new hybrid molecules could ultimately overcome that snag. The 2-phenylbenzothiazole (PhBTA) moiety was herein selected following previous studies on TAC-benzothiazole hybrids with different types of linkers, in which the best AChEi activity was found for the corresponding 2-phenyl derivative [16]. In fact, benzothiazoles are scaffolds that have long been of recognized interest for CNS targeted drugs [17]. On the other hand, due to the high analogy with the Thioflavin T (ThT) marker of β-amyloid plaques, several 2-phenylbenzothiazole derivatives have been recently reported as new imaging agents of Aβ plaques [18,19]. Furthermore, a methoxy substituent was included, aimed at balancing the expected hydrophobic nature of the hybrids and also to provide a pro-chelating capacity by masking the corresponding hydroxyl derivatives, which could work as a metal chelator and also account for the improvement of the anti-oxidant properties. Following the design of the compounds, a set of six selected compounds (**14**–**19**) were synthesized and bioassayed for their multifaceted properties. The effect of different substituents and linker sizes in biological activity and the pharmacokinetic properties were evaluated by molecular simulation studies.

## 2. Results and Discussion

### 2.1. Molecular Design and Modelling

**Figure 1:**
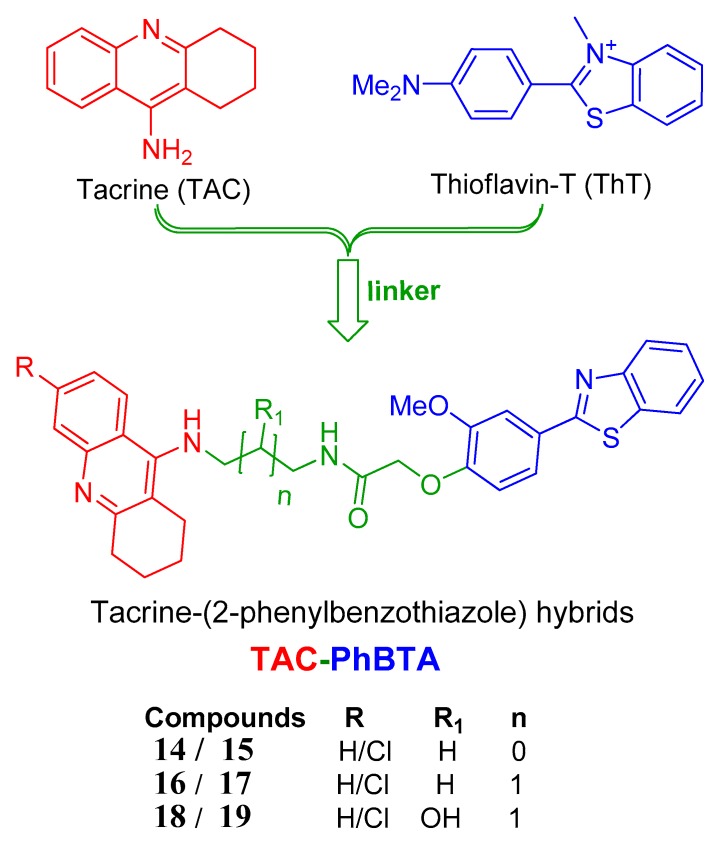
Design strategy of the tacrine-(2-phenylbenzothiazole (TAC-PhBTA) hybrids.

The strategy followed herein for the design of the hybrids includes conjugation of a known drug tacrine with the pharmacophore 2-phenylbenzothiazole, as shown in Figure 1, so that the conjugated molecules are able to inhibit the targeted AChE enzyme in a better way, along with other targets of AD such as Aβ_1–42_ self-aggregation.

Docking studies of these TAC-PhBTA hybrids into the active site of the known crystal structure of the *Torpedo californica* variant of the AChE enzyme (*Tc*AChE, obtained from PDB entry 1ODC) [20] were performed in order to know their binding mode and interactions. It is well known that the compounds which have capability for dual interactions with the catalytic triad (formed by three amino acids, Ser200, His440 and Glu327) and the peripheral active site (formed by Phe330, Trp84 and Glu199) of the enzyme have proved to be good AChE inhibitors [21]. Therefore, in aiming to find the optimal length of the alkyl spacer between the selected moieties of the designed inhibitors, it was decided to perform simulation studies into the active site of the *Tc*AChE enzyme with program GOLD v. 5.1 [22]; so that the hybrids can provide a dual-binding mode and thus improve AChE inhibition. The inhibitor *N*-quinolin-4-yl-*N*′-(1,2,3,4-tetrahydroacridin-9-yl)octane-1,8-diamine [20], obtained from the crystal structure of its complex with *Tc*AChE, was used as model ligand, as it also includes a tacrine moiety connected through a long carbon chain to an aminoquinoline moiety. The docking calculations were performed using the Astex Statistical Potential (ASP)scoring function, since this function has previously been proven to give the best docking predictions for AChE inhibitors [23,24]. The modeling work was performed with *Tc*AChE, instead of the Human variant *h*AChE, considering that the active site conservation is high and so differences between active sites should not have a big impact on the modelling studies. Furthermore there are more published X-ray structures of inhibitors complexed with *Tc*Ache than with *h*AChE, which also account for the general preferential use of *Tc*AChE in modeling studies.

**Figure 2:**
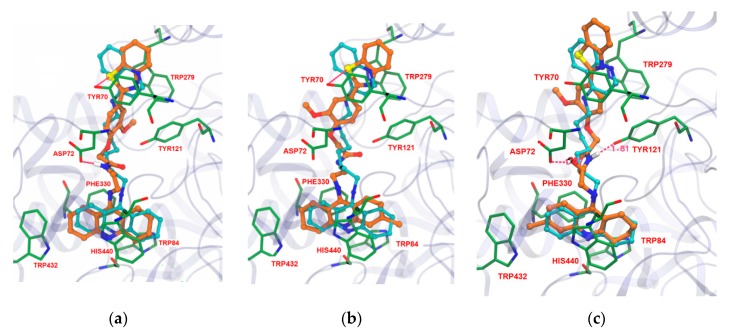
Docking results for the TAC-PhBTA hybrids with *Tc*AChE: Superimposition of **14**, **15**, and **19** (in brown colour of **a**, **b** and **c** respectively) with original ligand (sky blue).

The docking studies revealed that there are favourable interactions between the enzyme and the new inhibitors, with many similarities in their binding conformations to that of the original ligand. For example in Figure 2, compounds **14**, **15** and **19** show that the designed inhibitors are well inserted into the cavity of the active site, blocking the entrance to the substrate (acetylcholine) and water molecules. The TAC moiety is always found well implanted in the bottom of the gorge of the enzyme, binding to the CAS by π–π stacking with the aromatic ring of Trp84 and Phe330, overlapping almost perfectly with the TAC moiety of the original ligand. Generally, in the case of all synthesized TAC-PhBTA compounds, the alkyl spacer, along with the methoxyphenylbenzothiazole rings, seems to be well accommodated along the hydrophobic cavity and always placed at the entrance of the gorge. Their lipophilic nature, and the presence of ‘methoxy’, enables the binding with PAS, namely by aromatic stacking interaction with Tyr70 and Trp279, thus reinforcing the interactions with the enzyme. Moreover, all of the designed inhibitors showed one or two hydrogen bonding interactions with different amino acids. For example in compounds **14** and **15**, the sulfur atom of the benzothiazole ring establishes hydrogen bonds with Tyr70 while amidic NH or the carbonyl group of the linker were also found to exhibit hydrogen bonding interactions with Asp72 and Tyr121 in compounds **14** and **19**, therefore suggesting their strong fitting inside the cavity of the AChE enzyme. The docking simulation also indicates that insertion of a hydroxyl group in the linker chain (compound **19**) seems to enable extra H-bonding interaction with amino-acid residues (Asp72), Figure 2c.

### 2.2. Chemistry

**Scheme 1:**
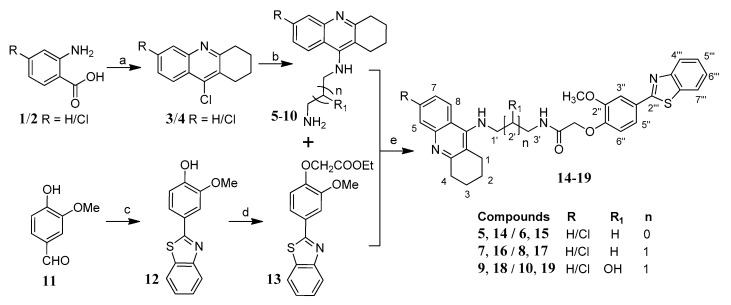
Synthesis of TAC-PhBTA hybrids; reagents and conditions: (a) cyclohexanone, POCl_3_, 180 °C, 3 h; (b) diaminoalkane (3.0 eq), phenol, KI, 165–170 °C 35–60 min; (c) 2-aminothiophenol, ethanol, reflux overnight; (d) DMF, K_2_CO_3_ (1.2 eq), ethylbromoacetate (1.05 eq), rt overnight; (e) MeOH, NH_4_Cl (catalytic), reflux 24–36 h.

The systematic approach for the synthesis of the designed tacrine-phenylbenzothiazole hybrids (TAC-PhBTA) is shown in Scheme 1. Firstly, alkyl substituted TAC-amines **5**–**10** of different chain lengths were synthesized according to the previously described method by our group [25]. Meanwhile, synthesis of ethyl 2-(4-(benzo[*d*]thiazol-2-yl)-2-methoxyphenoxy)acetate (**13**) was carried out by the reaction of ethylbromoacetate with 4-benzothiazol-2-yl-2-methoxy-phenol (**12**) which in turn was obtained by condensation reaction of 4-hydroxy-3-methoxy-benzaldehyde (**11**) with 2-aminothiophenol. Finally, condensation of different TAC-amines (**5**–**10**) with ethyl 2-(4-(benzo[*d*]thiazol-2-yl)-2-methoxyphenoxy)acetate (**13**) and a catalytic amount of ammonium chloride gave a good yield of the desired TAC-PhBTA hybrids.

### 2.3. Biological Studies

All of the newly synthesized TAC-PhBTA conjugates (**14**–**19**) were evaluated for the inhibition of AChE and Aβ_1–42_ self-aggregation activities through in vitro assays. Apart from biological studies, all of the studied hybrids were also investigated for pharmacokinetic properties by using the QikProp v. 2.5 [26] program which is provided by the MAESTRO v. 9.3 software package Schrodinger Inc., Portland, OR, USA) [27].

#### 2.3.1. Acetylcholinesterase Inhibition

In order to determine the inhibitory capacity of these hybrid compounds against the *Tc*AChE enzyme, a modified method of Ellman’s assay [28] was used and the results obtained are presented in Table 1. As expected from the docking simulation results, all of the designed hybrids displayed high inhibitory submicromolar activities against this enzyme (IC_50_ = 0.06–0.27 µM) and were found to be even better than the reference compound tacrine (IC_50_ = 0.30 µM) and other already studied TAC hybrids containing the BTA moiety, such as a TAC-BTA series without chloro substitution at the tacrine moiety (IC_50_ = 0.34–1.84 µM) [16] or a benzothiazole-hydroxypyridinone (BTA-HP) series (13.8 < IC_50_ > 500 µM) [29]. Nevertheless, some compounds of a series of tacrine-phenylbenzothiazole hybrids were reported with better inhibitory activity than those herein studied (IC_50_ = 0.017–0.105 µM) [16].

Among all the tested hybrids, compound **15**, with the ethylene chain linker and chloro substitution at the 6-position of the tacrine moiety, exhibited the best AChE inhibitory activity (IC_50_ = 0.06 μM). Further analysis of the obtained results allows concluding that compounds with chloro (Cl) substitution on the TAC moiety present better AChE inhibition then the corresponding unsubstituted analogues. This can be observed for the pairs of compounds **16**/**17**, with three carbon atoms in the linker, **14**/**15**, with two carbon atoms in the linker, or **18**/**19**, where a hydroxyl group has been further introduced in a three carbon linker chain. Among all the hybrids, those with a two carbon linker (compounds **14** and **15**) showed the best AChE inhibitory potential (IC_50_ = 0.06–0.11 µM), thus pointing towards an adequate length of two methylene groups to interact with both the CAS and PAS binding sites of the enzyme. The introduction of the hydroxyl group in the linker (**18** and **19**) did not result in improvement of the inhibitory activity, as compared with the corresponding non-substituted analogues (**16** and **17**).

Finally, although not considered under the framework of this paper, the inhibition of the butylcholinesterase (BuChE) and concomitant evaluation on the selectivity of both cholinesterases could be also of interest to be pursued in further future evaluations.

#### 2.3.2. Inhibition of Amyloid β Self-Aggregation

In order to determine the inhibitory potential of these hybrids (**14**–**19**) towards amyloid β_1–42_ self-aggregation, the in vitro approach relied on fluorescence emission by using the thioflavin T (ThT) assay and the results were compared with those of the previously known drug tacrine (see Table 1). Since the compounds of this series presented some solubility problems in methanol, the assays were performed with ligands at concentrations of 40 µM, i.e., half of the concentration usually used (80 µM) in these assays. Therefore, reference compound TAC was evaluated both at 80 µM and 40 µM, in order to compare with the obtained values of ligands at 40 µM. All of these compounds (**14**–**19**) show percent of inhibition values for Aβ self-aggregation in the range 27–44.6%, while tacrine presented a value of 11% that was independent of the value of the ligand concentration. From the obtained results it is possible to verify that all the TAC-BTA hybrids are moderate inhibitors of amyloid β_1–42_ self-aggregation, although better than the reference compound TAC. Among these hybrids, **14** (Aβ inhibition 44.6%), with a two carbon linker between TAC and BTA moieties and without Cl substitution in TAC, seems to allow the best interaction of the BTA moiety with the β-sheet secondary structure of amyloid-beta aggregates. However, Cl substitution on the phenyl ring of TAC (**15**) with the same chain length of the linker was found to produce a lower inhibitory capacity in comparison to **14**. A similar trend was observed for compounds with three carbon chain linkers, i.e., **17** vs. **16**, while further hydroxyl group introduction in the three carbon linker attenuated differences in the inhibitory capacity for the pair **18**/**19** (ca 31% of Aβ inhibition). So, it appears that introduction of a chloro substituent in TAC and/or a hydroxyl group in the carbon linker are not well suited for the interaction with β-sheet secondary structure of Aβ aggregates. But still all these hybrids are better inhibitors of Aβ aggregation than tacrine, so we can say that these compounds, composed of biaryl heterocyclic groups, are able to recognize and interact with the abnormal β-sheet conformation of the Aβ peptide and to induce the inhibition of fibril genesis. Similar conclusions were already achieved for other series of BTA-containing hybrids (TAC-BTA 40.7–51.8% [30], 22.3–61.3% [9] and hydroxypyridinone-BTA 19.7–68.9%) [29].

#### 2.3.3. Neuroprotective Effect of Tacrine-Phenylbenzothiazole Hybrids

**Figure 3:**
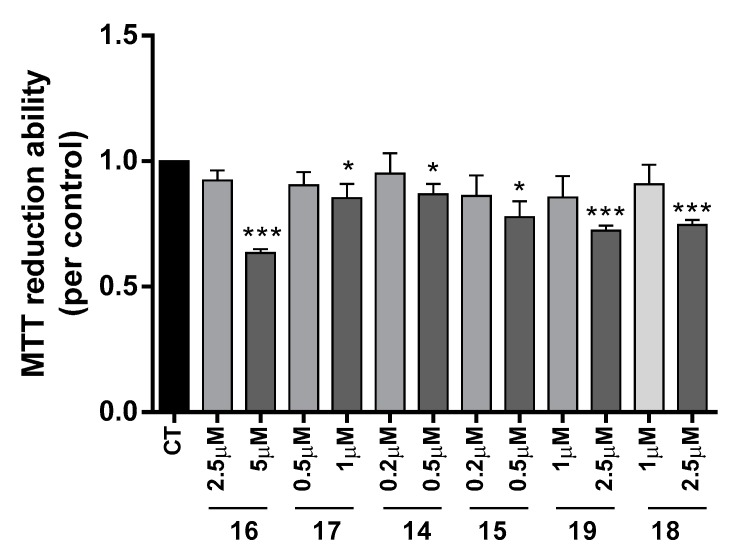
Dose-response screening to selected non-toxic concentrations of TAC-PhBTA. Cells were treated with varying concentration of TAC-PhBTA conjugates (from 0.2 μM to 5 μM) for 25 h. Evaluation of cell viability was performed by using MTT reduction assay. Results are expressed as the percentage of SH-SY5Y untreated cells, with the mean ± S.E.M. derived from 3 different experiments. * *p* < 0.05; *** *p* < 0.001, significantly different when compared with SH-SY5Y untreated cells.

The neuroprotective effect of TAC-PhBTA conjugates was evaluated using SH-SY5Y cells treated with Aβ_1–42_ peptides or ascorbate/iron. For each compound, a dose-response curve was performed to select a non-toxic concentration in order to analyze its neuroprotective effect (Figure 3).

**Figure 4:**
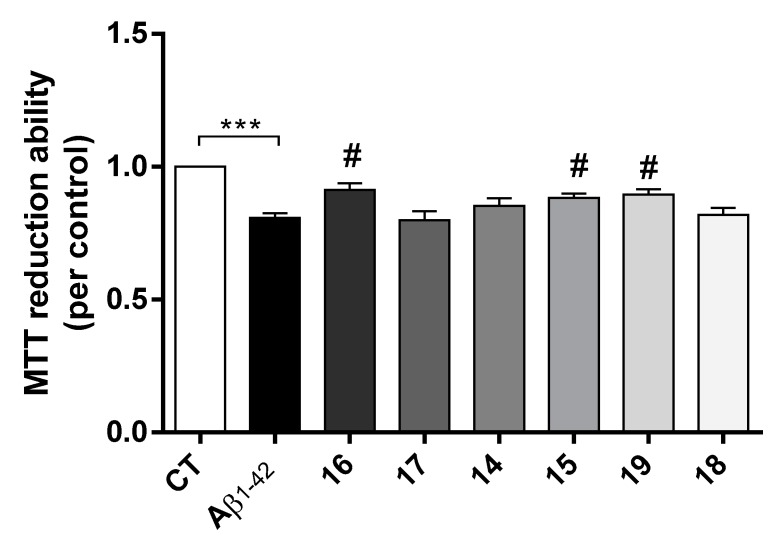
Neuroprotective effect of TAC-PhBTA conjugates on Aβ_1_-_42_ induced toxicity on SH-SY5Y cells. Cells were treated with Aβ_1_-_42_ peptide (1 μM), for 24 h after treatment for 1 h in the absence or the presence of the compounds. Evaluation of cell viability was performed using the MTT reduction assay. Results are expressed as the percentage of SH-SY5Y untreated cells, with the mean ± S.E.M. derived from 3 different experiments. *** *p* < 0.001, significantly different when compared with SH-SY5Y untreated cells; ^#^ *p* < 0.05, significantly different when compared with Aβ_1_-_42_ treated SH-SY5Y cells. (compounds: **16**—2.5 µM; **17**—0.5 µM; **14** and **15**—0.2 µM; **19** and **18**—1 µM).

AD is characterized by the extracellular formation of senile plaques composed of aggregated amyloid-beta peptide (Aβ) along with dyshomeostasis of redox-active species. The production of ROS conjugated with oligomeric Aβ peptide are involved in the neurodegenerative process of AD [31]. Herein it was observed that Aβ induced a decrease in cell viability and, interestingly, compounds **16**, **15** and **19** prevented Aβ-induced cell toxicity (Figure 4).

**Figure 5:**
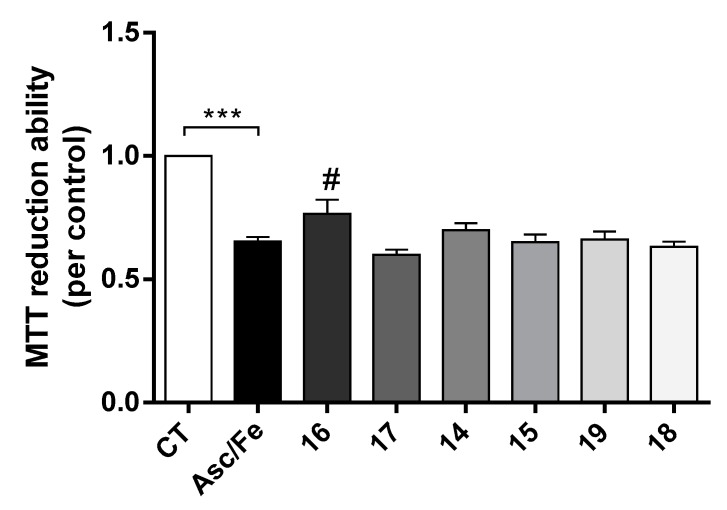
Neuroprotective effect of TAC-PhBTA conjugates against Ferrous Sulfate (Fe)/L-Ascorbic Acid (AscH(-)) toxicity on SH-SY5Y cells. Cells were treated with Fe/Asc (500 μM/5 mM, respectively) for 24 h, after treatment for 1h in the absence or the presence of the compounds. Evaluation of cell viability was performed by using MTT reduction assay. Results are expressed as the percentage of SH-SY5Y untreated cells, with the mean ± S.E.M. derived from 3 different experiments. *** *p* < 0.001, significantly different when compared with SH-SY5Y untreated cells. ^#^ *p* < 0.05, significantly different when compared with Fe/Asc treated SH-SY5Y cells (compounds: **16**—2.5 µM; **17**—0.5 µM; **14** and **15**—0.2 µM; **19** and **18**—1 µM).

Compounds **14**, **16**, **17** and **19** also showed the ability to inhibit Aβ-self-aggregation. Additionally, data gathered from the literature shows that oxidative stress plays a key role in the pathogenesis of AD [31]. Indeed, oxidative stress is an early event in the course of AD, associated with increased levels of protein, lipid, and nucleic acid oxidation in the hippocampus and cortex of AD patients that have elevated levels of Aβ_1-40_ and Aβ_1-42_. To mimic oxidative stress in our cell model we used the pair Asc/Fe. In fact, it was observed that Asc/Fe induced a decrease in cell viability in SHSY-5Y cells (Figure 5). However, among the new TAC-PhBTA hybrids tested, only compound **16** showed a statistical significant neuroprotective effect. These results show that compound **16,** which is soluble in methanol, has some radical scavenging properties.

#### 2.3.4. Pharmacokinetic Characterization

From the in silico pharmacokinetic study, parameters such as the lipo-hydrophilic character (clog *P*), the ability to cross the blood-brain barrier (log BB) and the ability to be absorbed from the intestinal tract to the blood (Caco-2 cell permeability), were calculated along with verification of Lipinski’s rule of five [32]. As it can be seen in Table 1, all the compounds present clog *P* (octanol/water) coefficients close to but slightly superior to five, which means that they have a lipophilic character higher than recommended by Lipinski’s rule. All of these compounds also have molecular weights higher than 500, even higher than 600 for **19**, which accounts for two violations of this rule. Concerning Caco-2 permeability, some compounds (**14**–**17**) exhibited very good results, ranging from ca 700-1086 nm/s (higher than 500 nm/s is considered good [26]), indicating that the absorption through the intestinal tract to the blood is possible. Nevertheless, compounds **18** and **19** have values lower than 500 nm/s which suggests lower absorption through intestinal tract to the blood. Finally, the high lipophilic character and the low blood-brain barrier permeability (log BB) provides a conclusion that all these compounds are not eligible drug candidates for oral administration, and require further improvement of the synthetic approach to ameliorate the pharmacokinetic properties, namely the compound absorption and entering into the cells.

## 3. Materials and Methods

### 3.1. Experimental

#### 3.1.1. Equipment/Reagents

The analytical grade reagents were purchased from Sigma-Aldrich (St. Louis, MO, USA) and Acros (Thermo Fisher Scientific, Geel, Belgium) and were used without any further purification. The organic solvents were dried and distilled according to standard methods described in literature [33]. The reactions were monitored on precoated thin layer chromatography (TLC) plates (Merck silica gel 60F 254) and the reaction spots were visualized either by UV light. Silica gel of mesh size 230–400 (Geduran Si 60, Merck, Kenilworth, NJ, USA), was used for column chromatographic separations/purifications of compounds. The Proton and Carbon-13 NMR were recorded either on Bruker AVANCE III-300 (300 MHz and 75.5 MHz) or Bruker AVANCE III-400 (400 MHz and 100.5 MHz) NMR spectrometers (Billerica, MA, USA) at 25 °C using peaks of solvents as internal standard. The chemical shift values of protons or carbons were measured on a δ scale and the coupling constant values (*J*) were determined in Hertz. In the NMR representation, abbreviations are mentioned as: singlet represented by s, doublet represented by d, triplet represented by t, multiplet represented by m, double doublet represented by dd, and broad singlet represented by brs. The Leica Galen III hot stage apparatus (Leica Microsystems, Wetzlar, Germany) was used for measuring the melting points of compounds. Elemental analyses were performed on a Fisons EA1108 C/H/N/O instrument (Thermo Scientific, Waltham, MA, USA) and were within the limit of ± 0.4%. The AChE inhibitory activity was measured in a 1 cm path length quartz cell through a Perkin Elmer Lambda 35 spectrophotometer equipped with a temperature programmer PTP1+1 Peltier System (T = 25.0 ± 0.1 °C) (Waltham, MA, USA). Electrospray ionization-mass (ESI-MS) measurements were carried out on a LCQ Fleet mass spectrometer (Thermo Scientific, S. Jose, CA, USA) operated in the ESI positive and negative ion modes.

#### 3.1.2. Acetylcholinesterase Assay

Modified Ellman’s method [28] was used as described previously for the determination of AChE activity. The AChE stock solution was prepared by dissolving commercially available enzyme AChE 500 U (extracted from *electrophorus electricus)* in 10 mL of TRIS buffer (50 mM, pH 8). HEPES buffer was used for the dilution of the enzyme to give the final AChE concentration. The assay solution consisted of 374 μL of 4-(2-hydroxyethyl)-1-piperazineethanesulfonic acid (Hepes) buffer (50 mM, pH 8.0), 476 μL of 3 mM bis(3-carboxy-4-nitrophenyl)disulfide (DTNB), 25 μL of AChE (type VI-S, from electric eel) stock solution, a variable volume (10–50 μL) of the compound’s stock solution (1 mg/mL of MeOH), and the necessary amount of methanol to attain the same volume of sample mixture (0.925 mL) in a 1 mL cuvette. Samples were left to react for 15 min and then the reaction was monitored at wavelength of 405 nm for 5 min, after adding 75 μL of 16 mM acetylthiocholine iodide (AChI) solution. Assays were run with a blank containing all the components except AChE, which was replaced by HEPES buffer. The velocities of the reaction were calculated as well as the enzyme activity. A control reaction was carried out using the sample solvent (methanol) in the absence of any tested compound, and it was considered as 100% activity. The percentage inhibition of the enzyme activity due to the presence of increasing test compound concentration was calculated by the following Equation (1):

(1) $$\%I=100-\left(\frac{v_{i}}{v_{0}}\times 100\right)$$

In which *v*_i_ is the initial reaction rate in the presence of inhibitor and *v*_0_ is the initial rate of the control reaction considered as 100% activity. The inhibition curves were obtained by plotting the percentage of enzymatic inhibition versus inhibitor concentration and a calibration curve was obtained from which the linear regression parameters were calculated.

#### 3.1.3. Aβ Aggregation Assay

Commercially available Aβ_1-42_ peptide was obtained from Aldrich and stored at −20 °C. It was treated with 1,1,1,3,3,3-hexafluoropropan-2-ol (HFIP) to avoid self-aggregation and reserved. HFIP pre-treated Aβ_1–42_ samples were re-dissolved in CH_3_CN/Na_2_CO_3_ (300 µM)/NaOH (250 µM) (48.3:48.3:4.3, *v*/*v*/*v*) solvent mixture in order to have a stable stock solution; this solution (500 µM) was further diluted in phosphate buffer (0.215 M, pH 8.0) to obtain 40 µM solution. The ligand, which was previously dissolved in methanol, was further diluted in phosphate buffer to a final concentration of 40 µM. Aβ_1_–_42_ aggregation was determined by measuring the fluorescence emission of Thioflavin T dye [34,35], with a Cary Eclipse Varian fluorimeter (Molecular Devices, San José, CA, USA).

After incubation for 24 h at 37 °C, without stirring, the samples were added to a 96-well plate (BD Falcon) with 180 µL of 5 µM ThT in 50 mM glycine-NaOH (pH 8.5) buffer. Blank samples were prepared for each concentration in a similar way, devoid of peptide. As for the control, a sample of the peptide was incubated under identical conditions but without the inhibitor. After 5-min incubation with the dye, the ThT fluorescence was measured at the following wavelengths: 446 nm (excitation) and 486 nm (emission). The percent inhibition of the self-induced aggregation due to the presence of the test compound was calculated by Equation (2), in which *IF_i_* and *IF_0_* corresponded to the fluorescence intensities, in the presence and the absence of the test compound, minus the fluorescence intensities due to the respective blanks.

*I*% = 100 − (*IF_i_*/*IF*_0_ × 100) (2)

The reported values were obtained as the mean ± S.E.M. of two different experiments.

#### 3.1.4. Cell Viability and Neuroprotection

SH-SY5Y human neuroblastoma cell line (ATCC-CRL-2266) grown in Dulbecco’s modified Eagle’s medium (DMEM) obtained from Gibco-Invitrogen (Life Technologies Ltd., Liverpool, UK) with 10% heat inactivated fetal calf serum, containing 50 U/mL penicillin, and 50 μg/mL streptomycin, under a humidified atmosphere of 95% air-5% CO_2_ at 37 °C. Cells were plated at 0.12 × 10^6^ cells/mL for cell viability assay. The tested compounds (**16, 17, 14, 15, 19** and **18)** were dissolved in DMSO at a concentration of 25 mM and aliquots were stored at −20 °C. We performed a dose-response screening (from 0.2 μM to 5 μM) in order to choose the highest non-toxic concentration. As a result we selected a 2.5 μM final concentration for compound **16**; 0.5 μM final concentration for compound **17**; 0.2 μM final concentration for compounds **14** and **15;** and a 1 μM final concentration for compounds **19** and **18**. The final concentration of DMSO in culture media did not exceed 0.05% (*v*/*v*) and no alterations to the cells were observed. The compounds were added to the cell media 1 h before the incubation with Aβ_1–42_ or ferrous sulfate/l-ascorbic acid. Aβ_1–42_ or ferrous sulfate/l-ascorbic acid where incubated alone or with the compound for an additional 24 h. Aβ_1–42_ was prepared as 276.9 μM stock in sterile water and added to the medium at 1 μM final concentration. Ferrous sulfate was freshly prepared as 0.36 M stock in water and added to the medium at 500 μM final concentration. l-Ascorbic Acid was freshly prepared as 80 mM stock in water and added to the medium at 5 mM final concentration. Aβ_1–42_ was purchased from Bachem (Torrance, CA, USA) and ferrous sulfate and L-ascorbic acid from Sigma Chemical Co (St. Louis, MO, USA).

Cell viability was determined by a MTT (3-(4,5-dimethylthiazol-2-yl)-2,5-diphenyltetrazolium bromide) reduction test. In viable cells, the enzyme succinate dehydrogenase metabolizes MTT into formazan that absorbs light at 570 nm. Following the cell treatment protocol the medium was aspirated and 0.5 mL MTT (0.5 mg/mL) was added to each well. The plate was then incubated at 37 °C for 1 h 30 min protected from light. At the end of the incubation period the formazan precipitates were solubilized with 0.5 mL of acidic isopropanol (0.04 M HCl/isopropanol). The absorbance was measured at 570 nm [36]. Cell reduction ability was expressed as a percentage of untreated control cells.

All data were expressed as mean ± S.E.M. of at least three independent experiments performed in duplicates. Statistical analyses were performed using one-way ANOVA followed by Bonferroni multiple comparisons procedure post hoc test. A *p* value < 0.05 was considered statistically significant.

#### 3.1.5. Molecular Modelling and Pharmacokinetic Properties

The X-ray crystallographic structure of acetylcholinesterase *Torpedo californica* AChE (*Tc*AChE) complexed with an inhibitor (original ligand), obtained from RCSB Protein Data Bank (PDB entry 1ODC) [18], was used to perform the docking simulations with the program GOLD v. 5.1 (CCDC Software Ltd, Cambridge, UK) [20]. For simulations, the original complex structure was treated using MAESTRO v. 9.3 (Schrodinger Inc., Portland, OR, USA) [27], by removing the original ligand and then 3D structures of the designed inhibitors were built with Maestro and their geometry was optimized by making a first random conformational search (RCS) of 1000 cycles and further optimized by 2500 optimization steps with the program GHEMICAL v. 2.0 (http://bioinformatics.org/ghemical/ghemical/index.html) [37]. Designed inhibitors were docked into the AChE using GOLD v. 5.1. with the default parameters of GOLD and the ASP scoring function.

The prediction of some pharmacokinetic properties, such as lipo-hydrophilic character (clog *P*), blood–brain barrier partition coefficient (log BB), the ability to be absorbed through the intestinal tract (Caco-2 cell permeability), were carried out using in silico tools, namely the program QIKPROP v. 2.5 (Schrodinger Inc., Portland, OR, USA) [26] provided by the MAESTRO software package [27].

#### 3.1.6. Synthesis

The schematic representation and the synthetic steps involved in the preparation of all tacrine-phenylbenzothiazole hybrids and their intermediates are shown in Scheme 1.

**General procedure for synthesis of *N^1^*-(1,2,3,4-tetrahydroacridin-9-yl)alkyl-diamines** (**5**–**10**): The mixture of 1,2,3,4-tetrahydroacridine [38] (**3**/**4**; 1 eq) and different diaminoalkanes was heated in phenol (0.5 eq) at 165–170 °C for a period of 35–60 min in the presence of potassium iodide in a catalytic amount. After the completion of reactions, monitored on TLC and on consumption of starting material, the reaction mixture was allowed to come at room temperature. After cooling, the reaction mixture was dissolved into DCM and washed with 5% NaOH (3 × 75 mL) and brine solution, respectively. Finally, the DCM layer so obtained was dried over the anhydrous Na_2_SO_4_ and then concentrated under reduced pressure to give desired product in crude form. The column purification of the crude product over silica gel (100–200 mesh) in 4–6% methanol/dichloromethane afforded the alkyl substituted tacrine amines (**5**–**10**) in a 60–75 % yield and their spectral data are presented in our previous publication [23].

**General procedure for synthesis of 4-(benzo[*d*]thiazol-2-yl)-2-methoxyphenol** (**12**): The mixture of vanillin (2 g, 0.086 mmol) and 2-aminothiophenol (0.090 mmol) in ethanol (25 mL) was heated to reflux overnight. After completion of the reaction, monitored on TLC and on full consumption of vanillin, reaction mixture was allowed to cool at 15 °C. The cooling of reaction to 15 °C leads to formation light yellow precipitates, which were filtered and washed with cold ethanol to give pure desired 4-(benzo[*d*]thiazol-2-yl)-2-methoxyphenol compound (**12**) in 95% yield; ^1^H-NMR (400 MHz, DMSO-*d*_6_), δ (ppm): 3.92 (s, 3H, OCH_3_), 6.98 (d, *J* = 8.0 Hz, 1H, H-6), 7.40 (t, 1H, *J* = 8.0 Hz, H-6′), 7.49–7.52 (m, 2H, H-5′ & H-5), 7.66 (s, 1H, H-3), 8.01–8.07 (m, 2H, H-4′ and H-7′); ^13^C-NMR (100.5 MHz, DMSO-*d*_6_), δ (ppm): 56.17, 110.58, 116.41, 121.78, 122.51, 122.79, 124.82, 125.36, 126.88, 134.69, 148.60, 150.61, 154.16, 168.04; *m/z* (ESI MS): calculated for C_14_H_12_NO_2_S (M + H)^+^: 258.058; obtained 258.06.

**General procedure for synthesis of ethyl 2-(4-(benzo[*d*]thiazol-2-yl)-2-methoxyphenoxy) acetate** (**13**): The mixture of 4-(benzo[*d*]thiazol-2-yl)-2-methoxyphenol (2 g, 7.77 mmol) and potassium carbonate (1.29 g, 9.32 mmol) in DMF (25 mL) was added with ethylchloroacate (1.14 g, 9.32 mmol) and the reaction mixture was stirred at 35 °C overnight. The progress of reaction was monitored on TLC and after completion, the solid residue of reaction mixture was filtered out and the mother liquid was concentrated to 5 mL volume under reduced pressure which on treatment with crushed ice gives white solid precipitates. Finally, filtration of precipitates under vacuum gives crude ethyl 2-(4-(benzo[*d*]thiazol-2-yl)-2-methoxyphenoxy) acetate (**13**), which by recrystallization with ethanol gives the white pure desired compound in 93% yield; ^1^H-NMR (400 MHz, DMSO-*d*_6_), δ (ppm): 1.20 (brs, 3H, CH_3_CH_2_OCO), 3.90 (s, 3H, OCH_3_), 4.16–4.17 (m, 2H, CH_3_CH_2_OCO), 4.87 (s, 2H, CH_2_O-), 7.01 (d, *J* = 8.0 Hz, 1H, H-6), 7.40–7.42 (m, 1H, H-6′), 7.50–7.55 (m, 2H, H-5′ & H-5), 7.66 (s, 1H, H-3), 8.00–8.07 (m, 2H, H-4′ and H-7′); ^13^C-NMR (100.5 MHz, DMSO-*d*_6_), δ (ppm): 14.52, 56.23, 61.24, 65.67, 110.46, 113.93, 121.10, 122.65, 123.04, 125.67, 127.01, 134.86, 149.75, 150.28, 154.08, 167.51, 168.87; *m/z* (ESI MS): calculated for C_18_H_18_NO_4_S (M + H)^+^: 344.095; obtained 344.11.

**General procedure for the synthesis of 2-(4-(benzo[*d*]thiazol-2-yl)-2-methoxyphenoxy)-*N*-(3-((1,2,3,4-tetrahydroacridin-9-yl)amino)alkyl)acetamide** (**14**–**19**): The mixture of ethyl 2-(4-(benzo[*d*]thiazol-2-yl)-2-methoxyphenoxy) acetate (13), (0.73 mmol) and tacrine amines (5–10) (0.91 mmol) in dry methanol (15 mL) was heated to reflux with a catalytic amount of ammonium chloride (0.01 mmol) under nitrogen atmosphere for 24–36 h. The progress of the reaction was monitored based on TLC and after the full consumption of benzothiazole ester (12), reaction was allowed to cool at room temperature which leads to precipitation of the desired product. The precipitates so obtained were filtered, washed with cold methanol and 0.1% HCl solution to remove the excess of tacrine amines. Finally, washing of precipitate with cold distilled water followed by diethyl ether washing gives the desired 2-(4-(benzo[*d*]thiazol-2-yl)-2-methoxyphenoxy)-*N*-(3-((1,2,3,4-tetrahydroacridin-9-yl)amino)alkyl)acetamide derivatives (14–19) in quantitative yields.

**2-(4-(Benzo[*d*]thiazol-2-yl)-2-methoxyphenoxy)-*N*-(2-((1,2,3,4-tetrahydroacridin-9-yl)amino)ethyl)acetamide** (**14**): M.p. = 99–101 °C, ^1^H-NMR (400 MHz, DMSO-*d*_6_), δ (ppm): 1.76 (brs, 4H, H-2 & H-3), 2.68 (brs, 2H, H-1), 2.86–2.87 (m, 2H, H-4), 3.34–3.36 (m, 2H, H-1′), 3.54–3.56 (m, 2H, H-2′), 3.88 (s, 3H, OCH_3_), 4.56 (s, 2H, OCH_2_CO), 5.59 (brs, 1H, N*H*), 6.95 (d, 2H, *J* = 8.0 Hz, H-6′′), 7.33 (t, 1H, *J* = 8.0 Hz, H-7), 7.43 (t, 1H, *J* = 8.0 Hz, H-5′′′), 7.47–7.54 (m, 3H, H-6, H-6′′′ and H-5′′), 7.65 (s, 1H, H-3′′), 7.70 (d, 1H, *J* = 8.0 Hz, H-5), 8.02 (d, 1H, *J* = 8.0 Hz, H-7′′′) 8.10-8.12 (m, 2H, H-8 and H-4′′′), 8.21 (brs, 1H, NH); ^13^C-NMR (100.5 MHz, DMSO-*d*_6_), δ (ppm): 22.14, 22.61, 24.81, 32.59, 39.66, 55.11, 68.37, 110.00, 114.67, 115.49, 119.63, 120.67, 121.48, 122.07, 122.85, 123.57, 125.07, 126.25, 126.35, 127.74, 128.48, 134.56, 146.16, 149.65, 149.89. 151.59, 153.53, 157.59, 167.94, 170.16; *m/z* (ESI MS): calculated for C_31_H_31_N_4_O_3_S (M + H)^+^: 539.21; obtained 539.43; elemental analysis calculated for (C_31_H_30_N_4_O_3_S • 0.7H_2_O): C 67.54, H 5.74, N 10.16, S 5.82%; found: C 67.45, H 5.69, N 10.22, S 5.75%.

**2-(4-(Benzo[*d*]thiazol-2-yl)-2-methoxyphenoxy)-*N*-(2-((6-chloro-1,2,3,4 tetrahydroacridin-9-yl)amino)ethyl)acetamide** (**15**): M.p. = 109–111 °C, ^1^H-NMR (400 MHz, MeOD-*d*_4_), δ (ppm): 1.84 (brs, 4H, H-2 & H-3), 2.59 (brs, 2H, H-1), 2.84 (brs, 2H, H-4), 3.66 (t, 2H, *J* = 8.0Hz, H-1′), 3.89-3.92 (m, 5H, H-2′ & OCH_3_), 4.58 (s, 2H, OCH_2_CO), 6.95 (d, 2H, *J* = 8.0 Hz, H-6′′), 7.33–7.42 (m, 3H, H-7, H-5′′′ and H-5′′), 7.50 (t, 1H, *J* = 8.0 Hz, H-6′′′) 7.57 (d, 1H, *J* = 4.0 Hz, H-3′′), 7.63 (d, 1H, *J* = 4.0 Hz, H-5), 7.93–7.95 (d, 2H, H-7′′′ & H-8), 8.19 (d, 1H, *J* = 8.0 Hz, H-4′′′); ^13^C-NMR (100.5 MHz, MeOD-*d*_4_), δ (ppm): 21.08, 21.91, 24.01, 30.18, 39.17, 55.18, 68.20, 109.98, 113.65, 114.51, 115.75, 120.51, 121.51, 122.10, 124.67, 125.15, 126.32, 134.49, 136.51, 142.63, 149.60, 149.89, 153.50, 154.13, 167.73. 170.75; *m/z* (ESI MS): calculated for C_31_H_30_ClN_4_O_3_S (M + H)^+^: 573.17; obtained 573.34; elemental analysis calculated for (C_32_H_32_N_4_O_3_S • 1.6H_2_O): C 66.09, H 6.10, N 9.63, S 5.51%; found: C 65.62, H 5.97, N 9.59, S 5.37%.

**2-(4-(Benzo[*d*]thiazol-2-yl)-2-methoxyphenoxy)-*N*-(3-((1,2,3,4-tetrahydroacridin-9-yl)amino)propyl)acetamide** (**16**):M.p. = 93–95 °C, ^1^H-NMR (400 MHz, MeOD-*d*_4_), δ (ppm): 1.83 (brs, 4H, H-2 & H-3), 1.87–1.91 (m, 2H, H-2′) 2.66 (brs, 2H, H-1), 2.86 (brs, 2H, H-4), 3.41 (t, 2H, *J* = 8.0 Hz, H-1′), 3.58 (t, 2H, *J* = 8.0 Hz, H-3′), 3.88 (s, 3H, OCH_3_), 4.57 (s, 2H, OCH_2_CO), 6.98 (d, 2H, *J* = 8.0 Hz, H-6′′), 7.33–7.50 (m, 4H, H-7, H-5′′′, H-6′′′ and H-5′′), 7.55 (t, 1H, *J* = 8.0 Hz, H-6), 7.63 (s, 1H, H-3′′), 7.67 (d, 1H, *J* = 8.0 Hz, H-5), 7.89–7.93 (d, 1H, *J* = 8.0 Hz, H-7′′′ and H-8), 8.10 (d, 1H, *J* = 8.0 Hz, H-4′′′); ^13^C-NMR (100.5 MHz, MeOD-*d*_4_), δ (ppm): 21.65, 22.30, 24.38, 30.30, 31.26, 36.06, 45.14, 55.15, 68.30, 110.06, 114.54, 118.65, 120.67, 121.48, 122.06, 123.44, 123.91, 124.22, 125.10, 126.28, 127.65, 129.60, 134.52, 143.98, 149.74, 149.90, 152.99, 153.50, 155.47, 167.89, 169.79; *m/z* (ESI MS): calculated for C_32_H_33_N_4_O_3_S (M + H)^+^: 553.22; obtained 553.45; elemental analysis calculated for (C_32_H_32_N_4_O_3_S • 1.6H_2_O): C 66.09, H 6.10, N 9.63, S 5.51%; found: C 65.62, H 5.97, N 9.59, S 5.37%.

**2-(4-(Benzo[*d*]thiazol-2-yl)-2-methoxyphenoxy)-*N*-(3-((6-chloro-1,2,3,4-tetrahydroacridin-9-yl)amino)propyl)acetamide** (**17**)**:** M.p. = 97–99 °C, ^1^H-NMR (400 MHz, MeOD-*d*_4_), δ (ppm): 1.91 (brs, 4H, H-2 & H-3), 1.97–2.00 (m, 2H, H-2′), 2.70 (brs, 2H, H-1), 2.90 (brs, 2H, H-4), 3.48 (t, 2H, *J* = 8.0 Hz, H-1′), 3.69 (d, 2H, *J* = 8.0 Hz, H-2′), 3.98 (s, 3H, OCH_3_), 4.64 (s, 2H, OCH_2_CO), 7.08 (d, 2H, *J* = 8.0 Hz, H-6′′), 7.39 (d, 1H, *J* = 8.0 Hz, H-7), 7.45 (t, 1H, *J* = 8.0 Hz, H-5′′′), 7.52–7.57 (m, 2H, H-5′′ & H-6′′′), 7.67 (s, 1H, H-3′′), 7.72 (s, 1H, H-5), 7.98–8.00 (m, 2H, H-7′′′ & H-8), 8.17 (d, 1H, *J* = 8.0 Hz, H-4′′′); ^13^C-NMR (100.5 MHz, MeOD-*d*_4_), δ (ppm): 22.41, 22.81, 25.13, 30.66, 33.52, 36.35, 45.48, 56.13, 68.18, 110.21, 114.33, 116.33, 118.59, 121.10, 122.57, 122.84, 124.09, 125.68, 125.86, 126.48, 127.02, 127.15, 133.30, 134.65, 147.51, 149.70, 150.18, 151.08, 153.71, 159.83, 167.70. 168.55; *m/z* (ESI MS): calculated for C_32_H_32_ClN_4_O_3_S (M + H)^+^: 587.187; obtained 587.40. elemental analysis calculated for (C_32_H_31_ClN_4_O_3_S • 1.0H_2_O): C 63.51, H 5.50, N 9.26, S 5.30%; found: C 63.43, H 5.49, N 9.29, S 5.17%.

**2-(4-(Benzo[*d*]thiazol-2-yl)-2-methoxyphenoxy)-*N*-(2-hydroxy-3-((1,2,3,4-tetrahydroacridin-9-yl)amino)propyl)acetamide** (**18**)**:** M.p. =118–120 °C, ^1^H-NMR (400 MHz, MeOD-*d*_4_), δ (ppm): 1.79 (brs, 4H, H-2 & H-3), 2.68 (brs, 2H, H-1), 2.90 (brs, 2H, H-4), 3.26–3.29 (m, 2H, H-1′), 3.53–3.56 (m, 2H, H-3′), 3.86 (brm, 1H, H-2′), 3.91 (s, 3H, OCH_3_), 4.64 (s, 2H, OCH_2_CO), 5.51 (brs, 1H, NH or OH), 6.42 (brs, 1H, OH or NH), 7.04 (d, 2H, *J* = 8.0 Hz, H-6′′), 7.42–7.45 (m, 2H, *J* = 8.0 Hz, H-7 & H-5′′′), 7.49–7.55 (m, 2H, H-6′′′ & H-5′′), 7.64 (d, *J* = 4.0 Hz, H-5′′), 7.64 (d, 1H, *J* = 8.0 Hz, H-3′′), 7.68 (t, 1H, *J* = 8.0 Hz, H-6), 7.80 (d, 1H, *J* = 8.0 Hz, H-5), 8.02 (d, 1H, *J* = 8.0 Hz, H-8), 8.10 (d, 1H, *J* = 8.0 Hz, H-7′′′), 8.20 (t, 1H, *J* = 4.0 Hz, NH), 8.27 (d, 1H, *J* = 8.0 Hz, H-4′′′); ^13^C-NMR (100.5 MHz, MeOD-*d*_4_), δ (ppm): 21.89, 22.52, 24.54, 31.59, 42.66, 51.38, 56.20, 68.18, 68.96, 110.22, 114.44, 118.65, 121.11, 122.61, 122.90, 124.19, 124.54, 125.83, 127.03, 127.14, 130.34, 134.67, 143.74, 149.77, 150.24, 152.96, 153.79, 155.54, 167.57, 168.64; *m/z* (ESI MS): calculated for C_32_H_33_N_4_O_4_S (M + H)^+^: 569.22; obtained 569.41; elemental analysis calculated for (C_32_H_32_N_4_O_4_S • 0.8H_2_O): C 65.91, H 5.81, N 9.61, S 5.50%; found: C 65.62, H 5.97, N 9.59, S 5.37%.

**2-(4-(Benzo[*d*]thiazol-2-yl)-2-methoxyphenoxy)-*N*-(3-((6-chloro-1,2,3,4-tetrahydroacridin-9-yl)amino)-2-hydroxypropyl)acetamide** (**19**): mp = 112–115 °C; ^1^H-NMR (400 MHz, DMSO-*d*_6_), δ (ppm): 1.78 (brs, 4H, H-2 & H-3), 2.69 (brs, 2H, H-1), 2.87 (brs, 2H, H-4), 3.24–3.25 (m, 2H, H-1′), 3.44 (brm, 2H, H-3′), 3.74 (brs, 1H, H-2′), 3.91 (s, 3H, OCH_3_), 4.62 (s, 2H, OCH_2_CO), 5.32 (brs, 1H, NH or OH), 5.41 (brs, 1H, OH or NH), 7.03 (d, 2H, *J* = 8.0 Hz, H-6′′), 7.31 (d, 1H, *J* = 8.0 Hz, H-7), 7.44 (t, 1H, *J* = 8.0 Hz, H-5′′′), 7.51–7.53 (m, 2H, H-5′′ & H-6′′′), 7.67 (s, 1H, H-3′′), 7.71 (s, 1H, H-5), 8.02–8.14 (m, 4H, H-7′′′, H-8, H-4′′′ and NH); ^13^C-NMR (100.5 MHz, DMSO-*d*_6_), δ (ppm): 22.71, 23.01, 25.05, 33.97, 42.99, 52.02, 56.21, 68.37, 69.42, 110.30, 114.49, 116.63, 118.96, 121.11, 122.64, 123.03, 123.94, 125.69, 125.95, 127.02, 132.97, 134.85, 148.04, 149.88, 150.50, 151.09, 154.04, 159.76, 167.46, 168.34; *m/z* (ESI MS): calculated for C_32_H_32_ClN_4_O_4_S (M + H)^+^: 603.18; obtained 603.45; elemental analysis calculated for (C_32_H_31_ClN_4_O_4_S • 0.4H_2_O): C 62.97, H 5.25, N 9.18, S 5.25%; found: C 63.01, H 5.29, N 9.13, S 5.32%.

## 4. Conclusions

A series of tacrine-phenylbenzothiazole (TAC-PhBTA) hybrids were designed by conjugating an active AChE inhibitor (tacrine) with a benzothiazole moiety through an alkyl chain with adequate length capable of assuring a dual-binding mode of enzyme inhibition. The biological screening results showed that all of the designed compounds exhibit excellent AChE inhibitory activities, even better than the previous standard drug tacrine, with IC_50_ values lying in the sub-micromolar range (0.06–0.27 μM), and displayed moderate inhibition values for Aβ self-aggregation (27–44.6%). According to the molecular modelling results, which anticipated a good fitting of compounds **14** and **15** between CAS and PAS sites of AChE enzyme, due to their tendency to form H-bonding, this was confirmed by AChE inhibition assays (**14** IC_50_ = 0.11 μM and **15** IC_50_ = 0.06 μM). The in silico pharmacokinetic study showed that the compounds are not eligible drug candidates for oral administration and therefore require co-formulations or non-oral administration for bioavailability enhancement, to improve the rate and extent of the compound that is absorbed in the systemic circulation and reach the target site. Nevertheless, regarding the cell viability tests and the overall effects against Aβ aggregation and AChE inhibition of the newly developed TAC-PhBTA conjugates, it can be concluded that compounds **15** and **16** are the most promising. A new era is emerging regarding AD therapeutics and this class of TAC-PhBTA hybrids represents undoubtedly a worthwhile stake in the research towards multi-target-directed anti-AD drugs. In particular further studies are expected to be pursued, namely in terms of evaluation of their buthylcholinesterase inhibitory capacity as well as the synthetic strategy towards the replacement of the methoxy group by a hydroxyl substituent.

## Figures and Tables

**Table 1:** Summary of activities of TAC-PhBTA derivatives (**14**–**19**) towards the inhibition of AChE and Aβ_1–42_ self-aggregation as well as some predicted pharmacokinetic parameters ^a^.

Comp	R	R_1_	n	Inhibition of AChE (IC_50_, µM) ^b^	Inhibition of Aβ-self-aggregation (%) ^c,d^	MW	clog *P* ^e^	log BB ^f^	Caco-2 Permeability (nm/sec)
**14**	**H**	**H**	0	0.11	44.6	538.7	6.200	−0.927	1086
**15**	**Cl**	**H**	0	0.06	27.0	573.1	5.445	−0.914	722
**16**	**H**	**H**	1	0.15	33.7	552.7	6.237	−0.975	1057
**17**	**Cl**	**H**	1	0.13	32.5	587.1	6.726	−0.739	1022
**18**	**H**	**OH**	1	0.27	31.1	568.6	5.222	−1.592	388
**19**	**Cl**	**OH**	1	0.14	31.3	603.1	5.692	−1.268	496
**TAC**	-	-	-	0.30	11	198.1	-	-	-

^a^ Predicted values using program QikProp v. 2.5 [26]; ^b^ AChE from electric eel, IC_50_, inhibitor concentration (means of two experiments) for 50% inactivation of AChE; ^c^ Inhibition of self-mediated Aβ_42_ aggregation (means of two experiments). The Thioflavin-T fluorescence method was used, and the measurements were carried out in the presence of an inhibitor; ^d^ Assays performed with *C*_L_ = 40 μM; ^e^ Calculated octanol/water partition coefficient; ^f^ Brain/blood partition coefficient.
